# Supplementary material for: Modeling glioblastoma heterogeneity as a dynamic network of cell states
Source: Mol Syst Biol. 2021 Sep 16;17(9):e10105. doi: 10.15252/msb.202010105 (PMC8444284; doi:10.15252/msb.202010105)
Supplement: Supplementary file 6 — Source Data for Figure 5 [file MSB-17-e10105-s004.zip › Figure5A_sourcedata/GSEA_3071/hallmarks_stateIII.GseaPreranked.1623742540963/HALLMARK_EPITHELIAL_MESENCHYMAL_TRANSITION.html]

Details for gene set HALLMARK\_EPITHELIAL\_MESENCHYMAL\_TRANSITION[GSEA]

|  || Dataset | state33071 |
| Phenotype | NoPhenotypeAvailable |
| Upregulated in class | na\_neg |
| GeneSet | HALLMARK\_EPITHELIAL\_MESENCHYMAL\_TRANSITION |
| Enrichment Score (ES) | -0.5595204 |
| Normalized Enrichment Score (NES) | -3.5323927 |
| Nominal p-value | 0.0 |
| FDR q-value | 0.0 |
| FWER p-Value | 0.0 |
Table: GSEA Results Summary

  

Fig 1: Enrichment plot: HALLMARK\_EPITHELIAL\_MESENCHYMAL\_TRANSITION      
 Profile of the Running ES Score & Positions of GeneSet Members on the Rank Ordered List

  

| PROBE | GENE SYMBOL | GENE\_TITLE | RANK IN GENE LIST | RANK METRIC SCORE | RUNNING ES | CORE ENRICHMENT || 1 | LGALS1 |  |  | 230 | 0.323 | -0.0551 | No |
| 2 | MYL9 |  |  | 293 | 0.293 | -0.0667 | No |
| 3 | TPM4 |  |  | 306 | 0.290 | -0.0652 | No |
| 4 | TAGLN |  |  | 407 | 0.257 | -0.0873 | No |
| 5 | THY1 |  |  | 472 | -0.253 | -0.1000 | No |
| 6 | MYLK |  |  | 479 | -0.253 | -0.0976 | No |
| 7 | PLOD2 |  |  | 521 | -0.255 | -0.1042 | No |
| 8 | NTM |  |  | 553 | -0.257 | -0.1082 | No |
| 9 | BDNF |  |  | 806 | -0.270 | -0.1700 | No |
| 10 | SNTB1 |  |  | 821 | -0.270 | -0.1693 | No |
| 11 | RHOB |  |  | 865 | -0.272 | -0.1762 | No |
| 12 | PMP22 |  |  | 1023 | -0.281 | -0.2129 | No |
| 13 | PRRX1 |  |  | 1032 | -0.281 | -0.2105 | No |
| 14 | LAMC1 |  |  | 1120 | -0.286 | -0.2288 | No |
| 15 | TFPI2 |  |  | 1128 | -0.286 | -0.2260 | No |
| 16 | FSTL1 |  |  | 1254 | -0.293 | -0.2541 | No |
| 17 | IL15 |  |  | 1334 | -0.297 | -0.2701 | No |
| 18 | SLIT2 |  |  | 1400 | -0.300 | -0.2823 | No |
| 19 | WNT5A |  |  | 1531 | -0.308 | -0.3115 | No |
| 20 | PLOD3 |  |  | 1588 | -0.311 | -0.3212 | No |
| 21 | MSX1 |  |  | 1715 | -0.320 | -0.3491 | No |
| 22 | COL12A1 |  |  | 1764 | -0.322 | -0.3565 | No |
| 23 | NT5E |  |  | 1769 | -0.323 | -0.3524 | No |
| 24 | ID2 |  |  | 1780 | -0.323 | -0.3499 | No |
| 25 | LAMA2 |  |  | 1786 | -0.324 | -0.3460 | No |
| 26 | P3H1 |  |  | 1846 | -0.327 | -0.3563 | No |
| 27 | MATN3 |  |  | 1891 | -0.329 | -0.3625 | No |
| 28 | COL5A3 |  |  | 1895 | -0.329 | -0.3581 | No |
| 29 | TIMP1 |  |  | 2038 | -0.337 | -0.3899 | No |
| 30 | PLOD1 |  |  | 2056 | -0.338 | -0.3889 | No |
| 31 | PPIB |  |  | 2111 | -0.342 | -0.3976 | No |
| 32 | PLAUR |  |  | 2181 | -0.347 | -0.4102 | No |
| 33 | PDLIM4 |  |  | 2346 | -0.361 | -0.4474 | No |
| 34 | GJA1 |  |  | 2362 | -0.362 | -0.4455 | No |
| 35 | ENO2 |  |  | 2366 | -0.363 | -0.4405 | No |
| 36 | GLIPR1 |  |  | 2390 | -0.364 | -0.4407 | No |
| 37 | COL7A1 |  |  | 2404 | -0.365 | -0.4383 | No |
| 38 | CD44 |  |  | 2471 | -0.371 | -0.4497 | No |
| 39 | ECM2 |  |  | 2491 | -0.373 | -0.4487 | No |
| 40 | MMP2 |  |  | 2527 | -0.375 | -0.4519 | No |
| 41 | FBN2 |  |  | 2528 | -0.375 | -0.4459 | No |
| 42 | FLNA |  |  | 2559 | -0.378 | -0.4477 | No |
| 43 | QSOX1 |  |  | 2574 | -0.380 | -0.4453 | No |
| 44 | ACTA2 |  |  | 2721 | -0.393 | -0.4773 | No |
| 45 | MMP14 |  |  | 2811 | -0.400 | -0.4942 | No |
| 46 | SFRP1 |  |  | 2839 | -0.402 | -0.4949 | No |
| 47 | ABI3BP |  |  | 2870 | -0.405 | -0.4963 | No |
| 48 | SERPINH1 |  |  | 2921 | -0.410 | -0.5028 | No |
| 49 | TGM2 |  |  | 2933 | -0.413 | -0.4991 | No |
| 50 | JUN |  |  | 2935 | -0.413 | -0.4928 | No |
| 51 | ITGAV |  |  | 2942 | -0.414 | -0.4877 | No |
| 52 | SLC6A8 |  |  | 3004 | -0.420 | -0.4970 | No |
| 53 | TNFAIP3 |  |  | 3088 | -0.430 | -0.5119 | No |
| 54 | APLP1 |  |  | 3156 | -0.439 | -0.5225 | No |
| 55 | COL16A1 |  |  | 3197 | -0.442 | -0.5259 | No |
| 56 | HTRA1 |  |  | 3242 | -0.450 | -0.5302 | No |
| 57 | TNFRSF11B |  |  | 3305 | -0.461 | -0.5391 | No |
| 58 | MEST |  |  | 3363 | -0.473 | -0.5465 | No |
| 59 | SPOCK1 |  |  | 3372 | -0.474 | -0.5410 | No |
| 60 | FSTL3 |  |  | 3409 | -0.482 | -0.5427 | No |
| 61 | SERPINE1 |  |  | 3474 | -0.499 | -0.5515 | Yes |
| 62 | GAS1 |  |  | 3494 | -0.505 | -0.5485 | Yes |
| 63 | DPYSL3 |  |  | 3498 | -0.507 | -0.5412 | Yes |
| 64 | MFAP5 |  |  | 3536 | -0.523 | -0.5425 | Yes |
| 65 | TGFBR3 |  |  | 3546 | -0.525 | -0.5365 | Yes |
| 66 | DKK1 |  |  | 3556 | -0.530 | -0.5304 | Yes |
| 67 | CALU |  |  | 3566 | -0.533 | -0.5242 | Yes |
| 68 | SERPINE2 |  |  | 3573 | -0.537 | -0.5172 | Yes |
| 69 | ITGB1 |  |  | 3574 | -0.537 | -0.5086 | Yes |
| 70 | COL8A2 |  |  | 3582 | -0.540 | -0.5018 | Yes |
| 71 | LRP1 |  |  | 3584 | -0.540 | -0.4935 | Yes |
| 72 | ITGA2 |  |  | 3589 | -0.542 | -0.4859 | Yes |
| 73 | CTHRC1 |  |  | 3591 | -0.544 | -0.4774 | Yes |
| 74 | DST |  |  | 3597 | -0.545 | -0.4700 | Yes |
| 75 | THBS1 |  |  | 3599 | -0.547 | -0.4616 | Yes |
| 76 | MATN2 |  |  | 3641 | -0.572 | -0.4632 | Yes |
| 77 | COL4A2 |  |  | 3664 | -0.586 | -0.4596 | Yes |
| 78 | COL6A2 |  |  | 3674 | -0.595 | -0.4524 | Yes |
| 79 | PDGFRB |  |  | 3678 | -0.598 | -0.4437 | Yes |
| 80 | DCN |  |  | 3723 | -0.633 | -0.4451 | Yes |
| 81 | COL5A2 |  |  | 3726 | -0.636 | -0.4355 | Yes |
| 82 | PMEPA1 |  |  | 3737 | -0.657 | -0.4276 | Yes |
| 83 | VCAN |  |  | 3741 | -0.660 | -0.4178 | Yes |
| 84 | FBLN1 |  |  | 3744 | -0.664 | -0.4078 | Yes |
| 85 | FZD8 |  |  | 3754 | -0.676 | -0.3993 | Yes |
| 86 | TIMP3 |  |  | 3755 | -0.677 | -0.3885 | Yes |
| 87 | PCOLCE |  |  | 3777 | -0.708 | -0.3827 | Yes |
| 88 | TGFBI |  |  | 3781 | -0.713 | -0.3721 | Yes |
| 89 | SCG2 |  |  | 3786 | -0.718 | -0.3617 | Yes |
| 90 | CDH11 |  |  | 3789 | -0.721 | -0.3507 | Yes |
| 91 | FBN1 |  |  | 3790 | -0.723 | -0.3391 | Yes |
| 92 | INHBA |  |  | 3801 | -0.746 | -0.3298 | Yes |
| 93 | EDIL3 |  |  | 3810 | -0.758 | -0.3198 | Yes |
| 94 | SPARC |  |  | 3811 | -0.759 | -0.3077 | Yes |
| 95 | BGN |  |  | 3812 | -0.761 | -0.2956 | Yes |
| 96 | COL5A1 |  |  | 3815 | -0.764 | -0.2839 | Yes |
| 97 | ELN |  |  | 3818 | -0.766 | -0.2722 | Yes |
| 98 | COL11A1 |  |  | 3828 | -0.788 | -0.2620 | Yes |
| 99 | COL4A1 |  |  | 3829 | -0.791 | -0.2493 | Yes |
| 100 | CDH2 |  |  | 3840 | -0.813 | -0.2389 | Yes |
| 101 | FN1 |  |  | 3850 | -0.842 | -0.2279 | Yes |
| 102 | CADM1 |  |  | 3856 | -0.851 | -0.2156 | Yes |
| 103 | PTX3 |  |  | 3858 | -0.855 | -0.2022 | Yes |
| 104 | LUM |  |  | 3861 | -0.870 | -0.1888 | Yes |
| 105 | TNC |  |  | 3874 | -0.951 | -0.1768 | Yes |
| 106 | POSTN |  |  | 3885 | -1.025 | -0.1631 | Yes |
| 107 | COL6A3 |  |  | 3900 | -1.171 | -0.1480 | Yes |
| 108 | COL1A1 |  |  | 3902 | -1.191 | -0.1293 | Yes |
| 109 | IGFBP3 |  |  | 3903 | -1.199 | -0.1101 | Yes |
| 110 | COL1A2 |  |  | 3906 | -1.278 | -0.0902 | Yes |
| 111 | IGFBP2 |  |  | 3917 | -1.404 | -0.0705 | Yes |
| 112 | COL3A1 |  |  | 3918 | -1.418 | -0.0478 | Yes |
| 113 | SPP1 |  |  | 3920 | -1.508 | -0.0240 | Yes |
| 114 | MGP |  |  | 3927 | -1.633 | 0.0005 | Yes |
Table: GSEA details [plain text format]

  

Fig 2: HALLMARK\_EPITHELIAL\_MESENCHYMAL\_TRANSITION: Random ES distribution      
 Gene set null distribution of ES for **HALLMARK\_EPITHELIAL\_MESENCHYMAL\_TRANSITION**
